# RCTE: A multi-class object detection framework for dental panoramic radiographs

**DOI:** 10.1371/journal.pone.0359630

**Published:** 2026-10-08

**Authors:** Jiayi Peng, Ji Liu, Yufeng Shen, Miao Yin, Changzheng Liu, Jie Zhou, Jia Liu, Ronghua Zhang, Qi Hong

**Affiliations:** 1 School of Stomatology, Xinjiang Medical University, Urumqi, Xinjiang, China; 2 College of Information Science and Technology, Shihezi University, Shihezi, Xinjiang, China; 3 The First Affiliated Hospital of Shihezi University, Shihezi, Xinjiang, China; 4 Key Laboratory of Intelligent Decision-Making for Frontier Security, Xinjiang Production and Construction Corps, Shihezi, Xinjiang, China; SUNY Upstate Medical University Hospital, UNITED STATES OF AMERICA

## Abstract

**Background:**

Dental panoramic radiographs play an important role in oral disease screening, computer-aided diagnosis, and clinical treatment planning. However, accurate multi-class detection remains challenging due to small lesion sizes, ambiguous anatomical boundaries, insufficient multi-scale feature representation, and missed detections of complex dental structures.

**Objective:**

This study aims to develop an accurate and efficient multi-class object detection framework for dental panoramic radiographs to improve the detection performance of small lesions, weak-boundary structures, and complex anatomical targets.

**Methods:**

A novel re-parameterized cross-scale attention-enhanced framework, named RCTE, was proposed based on the YOLOv8n detector. The proposed framework integrates three complementary components: a Cross-Scale Channel Transformer (CSCT) module for cross-scale contextual interaction among *P*_3_, *P*_4_, and *P*_5_ features, a RepNCSPELAN4-based Re-parameterized Feature Pyramid Fusion (RPF) structure for enhancing multi-scale feature aggregation, and a Multi-Scale EMA (MS-EMA) mechanism for feature recalibration before the detection head. Experiments were conducted on a publicly available dental panoramic radiograph dataset containing 11 categories of dental structures and lesions. Model performance was evaluated using Precision, Recall, F1-score, mAP50, mAP75, and mAP50–95.

**Results:**

Compared with the original YOLOv8n baseline, RCTE improved mAP50, mAP75, mAP50–95, and Recall by 2.55, 3.88, 2.40, and 5.10 percentage points, respectively. The proposed framework achieved better detection completeness and localization accuracy compared with other YOLO-based detectors. Furthermore, RCTE maintained real-time inference capability, demonstrating a favorable balance between detection accuracy and computational efficiency.

**Conclusion:**

The proposed RCTE framework effectively improves multi-class object detection performance in dental panoramic radiographs by enhancing cross-scale feature interaction, multi-scale feature fusion, and detection feature recalibration. This method provides a potential solution for computer-aided dental image analysis.

## Introduction

Dental panoramic radiographs are widely used in oral disease screening, clinical diagnosis, treatment planning, and postoperative evaluation. Compared with ordinary intraoral radiographs, panoramic radiographs provide a relatively complete view of the maxillofacial region, including teeth, mandible, maxilla, mandibular canal, maxillary sinus, implants, crowns, and other anatomical or pathological structures. Therefore, accurate interpretation of dental panoramic radiographs is important for assisting dentists in identifying dental diseases and structural abnormalities. However, manual interpretation is time-consuming and highly dependent on clinical experience. Previous studies have also emphasized that panoramic X-ray interpretation is prone to error and that artificial intelligence has the potential to improve dental diagnosis and treatment planning [1]. In addition, the visual appearance of different dental categories may be similar, and some lesions may have low contrast or ambiguous boundaries, which increases the difficulty of accurate diagnosis.

With the development of deep learning, object detection methods have been increasingly applied to medical image analysis and dental radiograph interpretation. In dental panoramic radiographs, deep learning-based detectors can automatically locate and classify multiple dental structures or lesions, such as caries, periapical lesions, missing teeth, root canal treatment, implants, mandibular canals, and maxillary sinuses. For example, deep learning object detection techniques have been used for detecting and diagnosing maxillary sinus lesions on panoramic radiographs [2], detecting and classifying radiolucent mandibular lesions [3], and detecting teeth on pediatric panoramic radiographs using YOLO-v5-based models [4]. In addition, the DENTEX benchmark further promotes abnormal tooth detection with dental enumeration and diagnosis on panoramic X-rays [1]. These studies indicate that deep learning-based detection methods provide an effective technical basis for computer-aided dental diagnosis.

Nevertheless, dental panoramic radiograph detection remains a challenging task. First, the scale difference among dental targets is significant. Some categories, such as caries and periapical lesions, usually occupy small local regions, while maxillary sinuses and mandibular canals may cover larger and more elongated areas. Second, many dental structures have blurred boundaries and low contrast with surrounding tissues, making accurate localization difficult. Third, anatomical structures often overlap in panoramic radiographs, which may lead to feature confusion and missed detections. Similar challenges have been observed in previous studies on automatic detection of dental lesions and anatomical structures in panoramic radiographs, where irregular shapes, low contrast, complex surrounding structures, and limited data may affect detection and localization performance [2,3,5]. These characteristics require detection models to have stronger multi-scale feature representation, contextual modeling, and feature discrimination capabilities.

YOLO-based detectors have been widely used in real-time object detection because of their simple structure, high inference speed, and favorable accuracy-efficiency balance. YOLOv8 further improves detection performance through an efficient backbone, feature pyramid structure, and anchor-free detection head [6–8]. Multi-scale feature fusion is essential for object detection, and Feature Pyramid Networks (FPNs) have shown that hierarchical convolutional features can be effectively used to construct high-level semantic feature maps at multiple scales [9,10]. However, when directly applied to dental panoramic radiographs, the original YOLOv8n still has several limitations. The conventional feature fusion structure may not sufficiently aggregate shallow spatial details and deep semantic information, which limits the detection of small lesions and weak-boundary targets. Moreover, the interactions among different feature scales are mainly achieved through top-down and bottom-up feature fusion, while explicit contextual dependencies among *P*_3_, *P*_4_, and *P*_5_ features are insufficiently modeled. As a result, the detector may fail to fully capture the global anatomical relationships in dental radiographs, especially for complex structures such as mandibular canals and maxillary sinuses.

This study proposes a novel re-parameterized cross-scale attention-enhanced framework named RCTE for multi-class object detection in dental panoramic radiographs. Built upon the YOLOv8n framework [6], RCTE improves the detector from three complementary perspectives: cross-scale contextual interaction, re-parameterized multi-scale feature fusion, and multi-scale feature recalibration. These designs aim to improve the detection of small lesions, elongated anatomical structures, and weak-boundary dental targets in panoramic radiographs.

The main contributions of this paper are summarized as follows:

- A re-parameterized cross-scale attention-enhanced detection network, named RCTE, is proposed for multi-class object detection in dental panoramic radiographs.
- A Cross-Scale Channel Transformer (CSCT) module is introduced to jointly model the *P*_3_, *P*_4_, and *P*_5_ feature maps, enhancing contextual information exchange across different feature levels.
- A RepNCSPELAN4-based Re-parameterized Feature Pyramid Fusion (RPF) structure is adopted to replace the conventional neck fusion units, strengthening multi-scale feature aggregation and information flow between shallow spatial details and deep semantic features.
- A Multi-Scale EMA (MS-EMA) feature recalibration mechanism is embedded before the detection head to enhance the model’s attention to low-contrast lesions and boundary-ambiguous regions at different detection scales.
- Experimental results show that RCTE improves mAP50, mAP75, mAP50–95, and Recall by 2.55, 3.88, 2.40, and 5.10 percentage points, respectively, compared with the original YOLOv8n, demonstrating the effectiveness of the proposed method.

## Related work

### Deep learning for dental panoramic radiograph analysis

Dental panoramic radiographs have been widely studied in computer-aided dental diagnosis because they provide a global view of teeth, jaws, and surrounding anatomical structures. In recent years, deep learning methods have shown strong potential in automatically detecting and localizing dental structures and pathological findings. Tuzoff et al. proposed a convolutional neural network-based method for tooth detection and numbering in panoramic radiographs, demonstrating the feasibility of automated dental charting from panoramic images [11]. Lee et al. evaluated a deep learning-based convolutional neural network for detecting and diagnosing dental caries on periapical radiographs [12]. Lee et al. further developed a deep learning model for early dental caries detection on bitewing radiographs and showed that CNN-based systems can support clinicians in caries diagnosis [13]. Mohammad-Rahimi et al. systematically reviewed deep learning methods for caries detection and reported the growing potential of deep learning in dental radiographic diagnosis [14].

Although these studies demonstrate the effectiveness of deep learning in dental radiograph analysis, accurate multi-class detection in panoramic radiographs remains challenging. Dental targets often vary greatly in scale and shape. Small lesions, such as caries and periapical lesions, usually occupy limited local regions, whereas mandibular canals and maxillary sinuses may appear as larger and elongated anatomical structures. Moreover, low contrast, blurred boundaries, and structural overlap may lead to missed detections and inaccurate localization. Therefore, a detector for dental panoramic radiographs should not only preserve fine spatial details but also model contextual relationships across different feature levels.

### Multi-scale feature fusion and cross-scale context modeling

Multi-scale feature representation is essential for object detection because objects may appear at different sizes and resolutions. Deep residual networks show that shortcut connections can improve gradient propagation and enable deeper feature learning [15]. Path Aggregation Network (PANet) further enhances information flow by adding a bottom-up path augmentation structure, indicating the importance of strengthening the propagation of localization information from lower layers to higher layers [16]. EfficientDet introduces a weighted bidirectional feature pyramid network (BiFPN) for efficient multi-scale feature fusion, showing that adaptive and bidirectional feature aggregation can improve detection efficiency and accuracy [17].

However, conventional feature fusion structures mainly rely on upsampling, downsampling, concatenation, and convolutional fusion. These operations can transfer information across feature levels, but they may be insufficient to explicitly model cross-scale contextual dependencies. For dental panoramic radiographs, this limitation is more evident because local lesions and global anatomical structures may be semantically related. For example, detecting a small lesion may require both local boundary details and surrounding anatomical context. Transformer-based models demonstrate that attention mechanisms can capture long-range dependencies and global contextual relationships [18]. Inspired by this idea, enhancing contextual interaction among different feature levels is meaningful for improving the detection of small lesions and complex anatomical structures in panoramic radiographs.

### Attention-based feature recalibration

Attention mechanisms have been widely introduced into convolutional neural networks to enhance feature discrimination. Squeeze-and-Excitation Networks (SENet) explicitly model channel-wise dependencies and adaptively recalibrate feature responses, providing a representative example of channel attention for feature enhancement [19]. Coordinate Attention further embeds positional information into channel attention by separately aggregating features along horizontal and vertical directions, which helps capture long-range dependencies while preserving location-sensitive information [20].

For dental panoramic radiographs, attention-based recalibration is particularly useful because many clinically important targets have low contrast, ambiguous boundaries, and irregular shapes. However, applying attention to only a single feature level may be insufficient. Different dental categories rely on different detection scales: small lesions require high-resolution spatial details, while larger anatomical structures require stronger semantic information. Therefore, multi-scale feature recalibration before the detection head can help improve the discriminative ability of fused detection features at different levels.

Based on the above observations, this study proposes RCTE by combining cross-scale contextual interaction, re-parameterized feature pyramid fusion, and multi-scale EMA-based feature recalibration. Compared with directly applying a general YOLOv8n detector, the proposed design aims to better address scale variation, weak boundaries, and feature confusion in multi-class dental panoramic radiograph detection.

## Materials and methods

### Overall architecture of RCTE

To improve multi-class object detection in dental panoramic radiographs, this study proposes RCTE, a re-parameterized cross-scale attention-enhanced detection network. The proposed model is developed based on the YOLOv8n framework and introduces three main improvements: a Cross-Scale Channel Transformer (CSCT) module, a RepNCSPELAN4-based Re-parameterized Feature Pyramid Fusion (RPF) structure, and a Multi-Scale EMA (MS-EMA) feature recalibration mechanism. The overall architecture of RCTE is shown in Fig 1.

**Fig 1:**
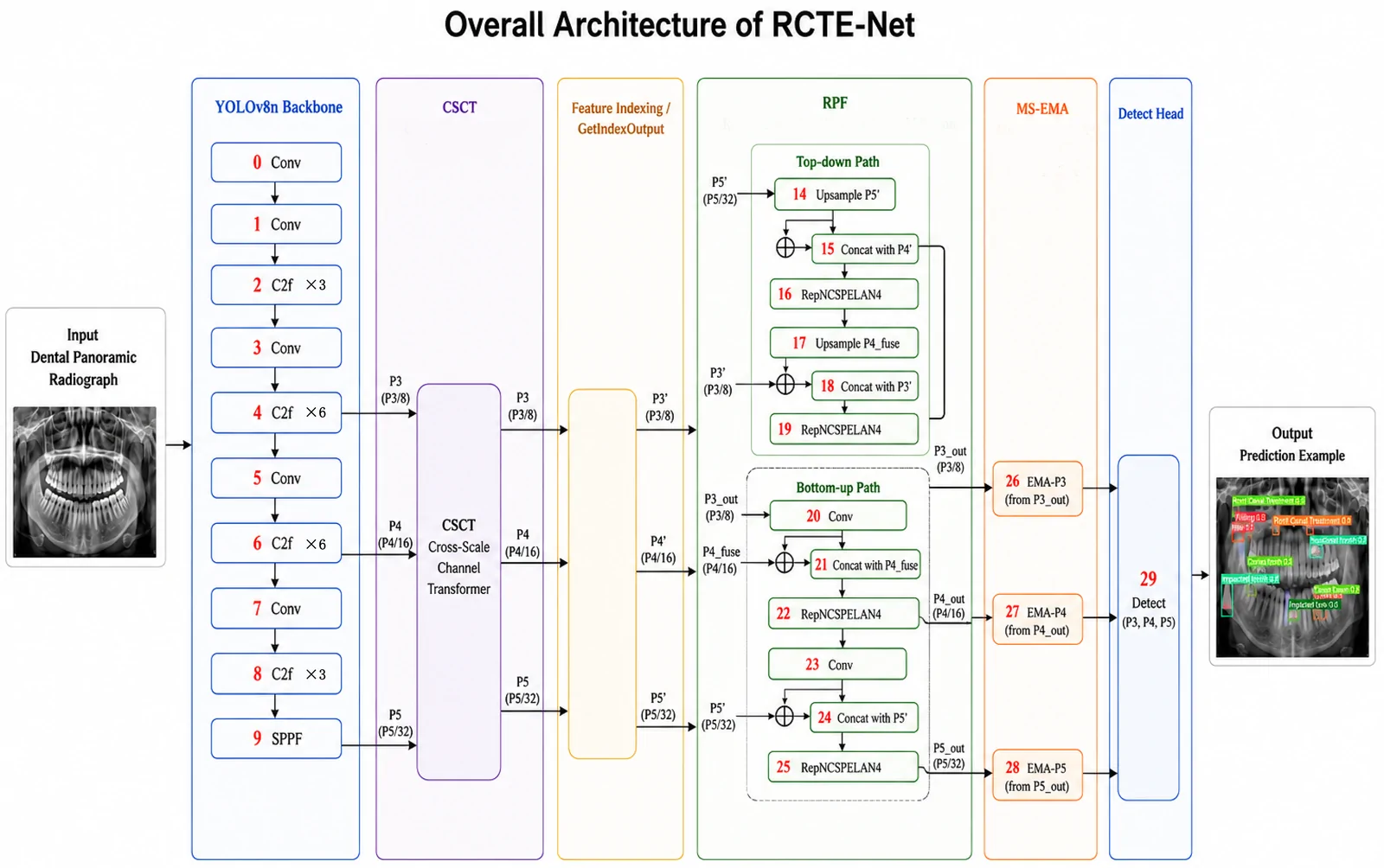
Overall architecture of RCTE. The proposed network consists of the YOLOv8n backbone, the CSCT module for cross-scale contextual interaction, the RPF structure for re-parameterized feature pyramid fusion, the MS-EMA module for multi-scale feature recalibration, and the original YOLOv8 detection head.

As shown in Fig 1, the proposed RCTE follows a progressive feature enhancement pipeline. The input dental panoramic radiograph is first processed by the YOLOv8n backbone to extract hierarchical features. Feature maps from layers 4, 6, and 9 are selected as *P*_3_, *P*_4_, and *P*_5_, respectively. These multi-scale features are first fed into the CSCT module for cross-scale contextual interaction. Then, the enhanced features are separated by feature indexing and passed to the RPF structure for bidirectional feature fusion. Finally, MS-EMA modules are applied to the fused features before the original YOLOv8 detection head.

### Cross-scale channel transformer module

A Cross-Scale Channel Transformer (CSCT) module is introduced after the backbone to enhance contextual interaction among multi-scale features, as shown in Fig 2. In the proposed network, the outputs corresponding to *P*_3_, *P*_4_, and *P*_5_ are simultaneously fed into the CSCT module. For the YOLOv8n setting, the channel dimensions of these three feature maps are 64, 128, and 256, respectively.

**Fig 2:**
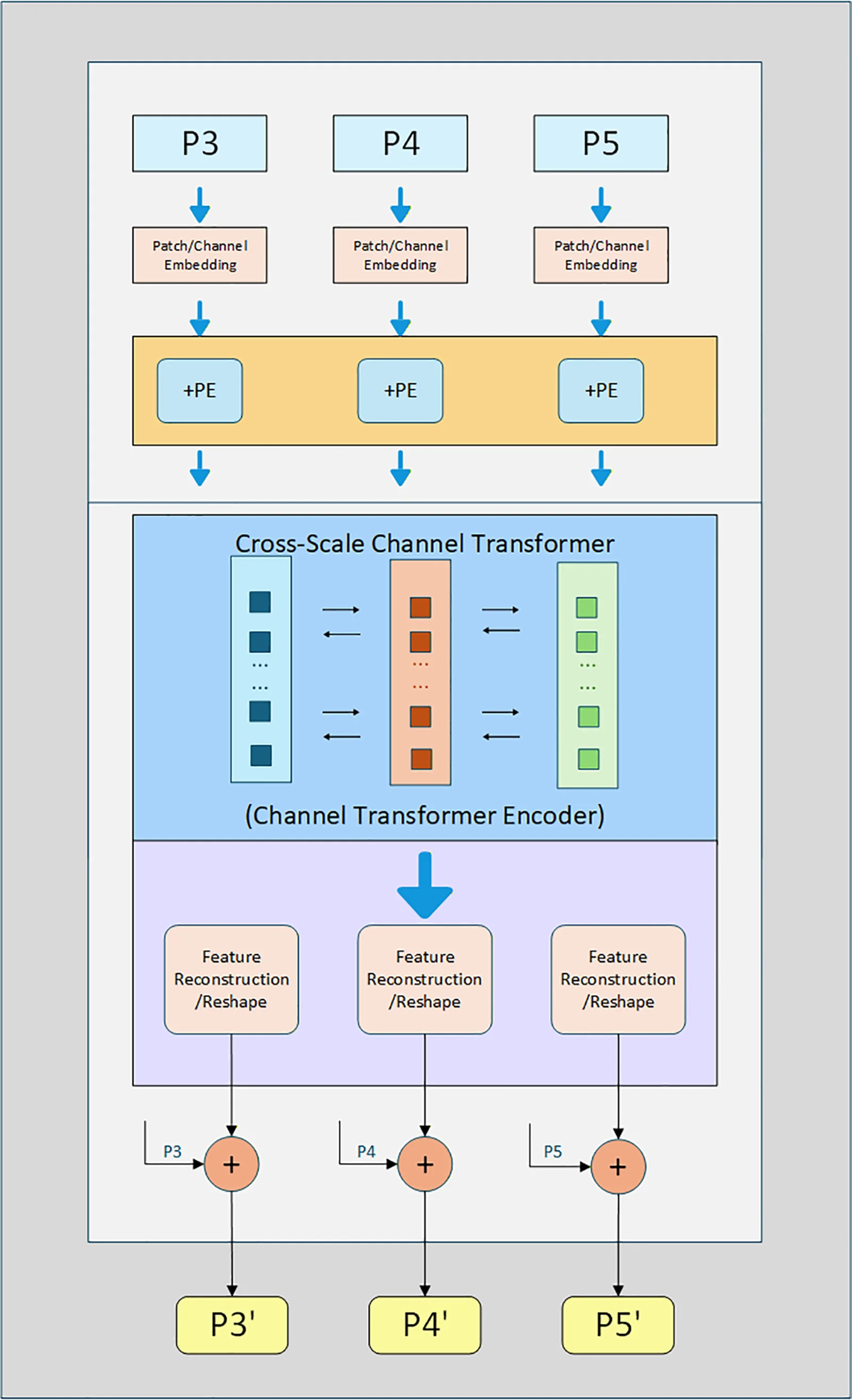
Structure of the proposed Cross-Scale Channel Transformer module. The CSCT module jointly models *P*_3_, *P*_4_, and *P*_5_ features to enhance contextual information exchange across different feature levels.

The CSCT module jointly models the multi-scale feature maps as follows:


$$\begin{array}{c} \{P_{3}^{\prime },P_{4}^{\prime },P_{5}^{\prime }\}=𝒯(P_{3},P_{4},P_{5}), \end{array}$$ (1)

where $P_{3}^{\prime }$, $P_{4}^{\prime }$, and $P_{5}^{\prime }$ denote the enhanced features after cross-scale interaction, and 𝒯(·) denotes the CSCT operation. In this module, each input feature map is first transformed into an embedded representation. Positional information is then added to preserve spatial structure. The embedded features from different scales are jointly processed through channel-wise Transformer interaction, allowing information exchange among shallow detailed features, middle-level structural features, and deep semantic features.

After Transformer-based interaction, the output tokens are reconstructed into feature maps and added to the corresponding input features. This residual reconstruction preserves the original feature representation while enhancing contextual information. Since the CSCT module outputs multiple enhanced feature maps, a feature indexing operation is used to separate $P_{3}^{\prime }$, $P_{4}^{\prime }$, and $P_{5}^{\prime }$. This operation only serves as an implementation-level feature extraction step and does not introduce additional complex computation.

Through CSCT, the contextual relationship among different scales is strengthened before feature pyramid fusion. This is beneficial for dental panoramic radiographs, where small lesions and large anatomical structures often coexist and require multi-scale contextual understanding.

### RepNCSPELAN4-based re-parameterized feature pyramid fusion

A RepNCSPELAN4-based Re-parameterized Feature Pyramid Fusion (RPF) structure is adopted to improve multi-scale feature aggregation in the neck. In the original YOLOv8n neck, C2f-based fusion units are used for feature fusion. In RCTE, these conventional fusion units are replaced by RepNCSPELAN4 modules to enhance feature reuse and strengthen the information flow between shallow spatial details and deep semantic features. The structure of the RepNCSPELAN4 block and its deployment in the top-down and bottom-up fusion paths are shown in Fig 3.

**Fig 3:**
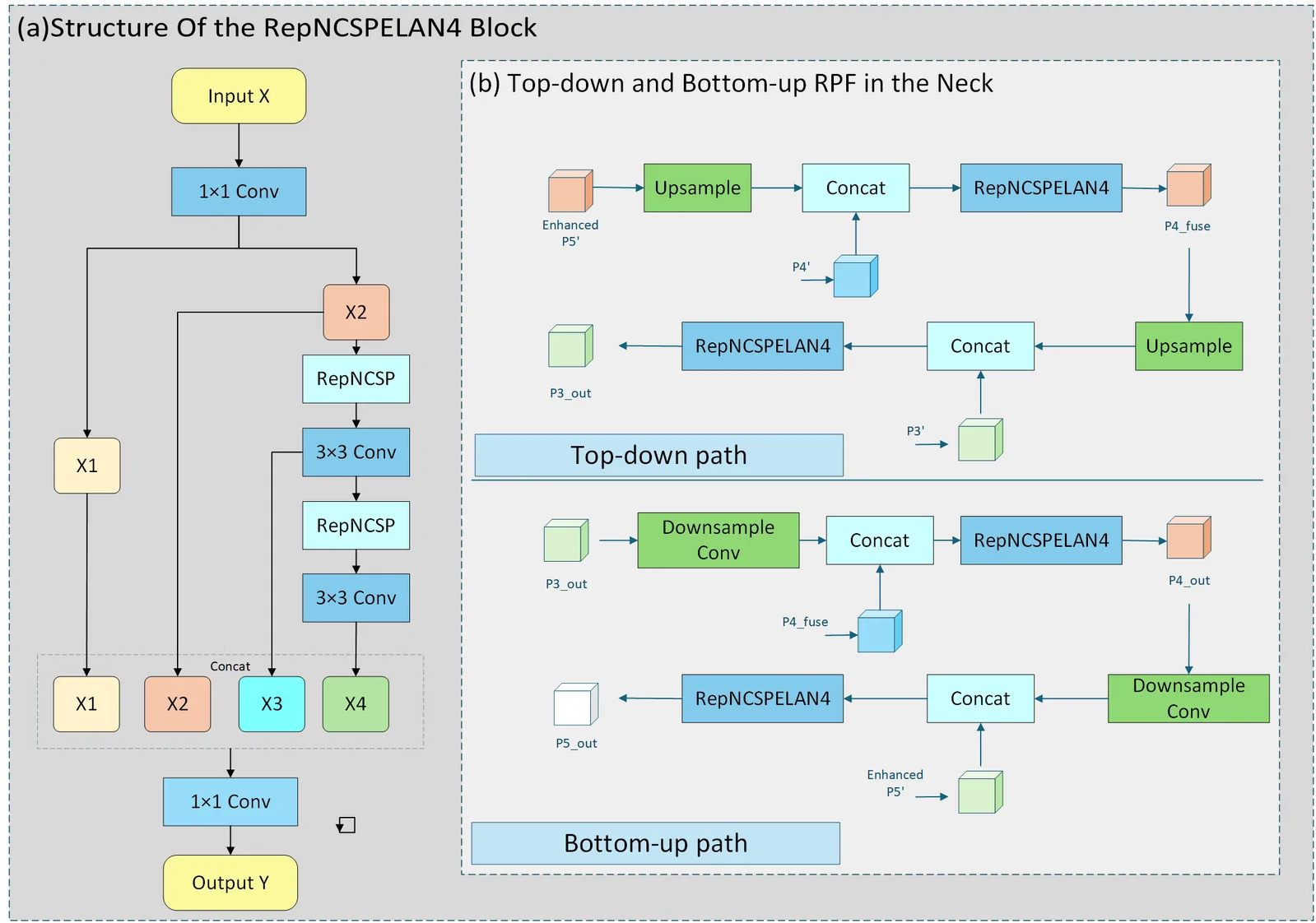
Structure of the proposed RepNCSPELAN4-based Re-parameterized Feature Pyramid Fusion module. (a) Internal structure of the RepNCSPELAN4 block. (b) Deployment of RepNCSPELAN4 modules in the top-down and bottom-up fusion paths for bidirectional multi-scale feature aggregation.

Given an input feature map *X*, the RepNCSPELAN4 module first performs channel projection using a 1×1 convolution:


$$\begin{array}{c} X_{0}={Conv}_{1\times 1}(X). \end{array}$$ (2)

Then, *X*_0_ is split into two parts along the channel dimension:


$$\begin{array}{c} X_{1},X_{2}=Split(X_{0}). \end{array}$$ (3)

One branch is retained as a shortcut feature, while the other branch is sequentially processed by RepNCSP-based convolutional operations:


$$\begin{array}{c} X_{3}={Conv}_{3\times 3}(RepNCSP(X_{2})), \end{array}$$ (4)

$$\begin{array}{c} X_{4}={Conv}_{3\times 3}(RepNCSP(X_{3})). \end{array}$$ (5)

Finally, the intermediate features are concatenated and fused by another 1×1 convolution:


$$\begin{array}{c} Y={Conv}_{1\times 1}(Concat(X_{1},X_{2},X_{3},X_{4})). \end{array}$$ (6)

Through this multi-branch design, feature reuse is enhanced and richer intermediate representations are preserved. Compared with conventional single-path fusion units, the RepNCSPELAN4 module facilitates more effective aggregation of shallow and deep features, which is beneficial for representing dental targets with complex shapes and ambiguous boundaries.

In the proposed RPF structure, RepNCSPELAN4 modules are deployed in both the top-down and bottom-up fusion paths of the neck. In the top-down path, high-level semantic features are progressively transmitted to lower-level features to enhance semantic representation. In the bottom-up path, localization-related information is further reinforced by aggregating lower-level spatial details into higher-level features. As a result, shallow spatial details and deep semantic information are more effectively integrated, thereby improving the representation of small lesions, elongated anatomical structures, and weak-boundary dental targets in panoramic radiographs.

### Multi-scale EMA feature recalibration

Although the RPF structure strengthens multi-scale feature fusion, the fused features may still contain redundant background responses and weak target activations. In dental panoramic radiographs, low-contrast lesions and ambiguous anatomical boundaries are easily affected by surrounding tissues, which may lead to missed detections or inaccurate localization. To further enhance the discriminative ability of the detection features, a Multi-Scale EMA (MS-EMA) feature recalibration mechanism is embedded before the detection head. The internal structure of the EMA block and its deployment on the three detection scales are shown in Fig 4.

**Fig 4:**
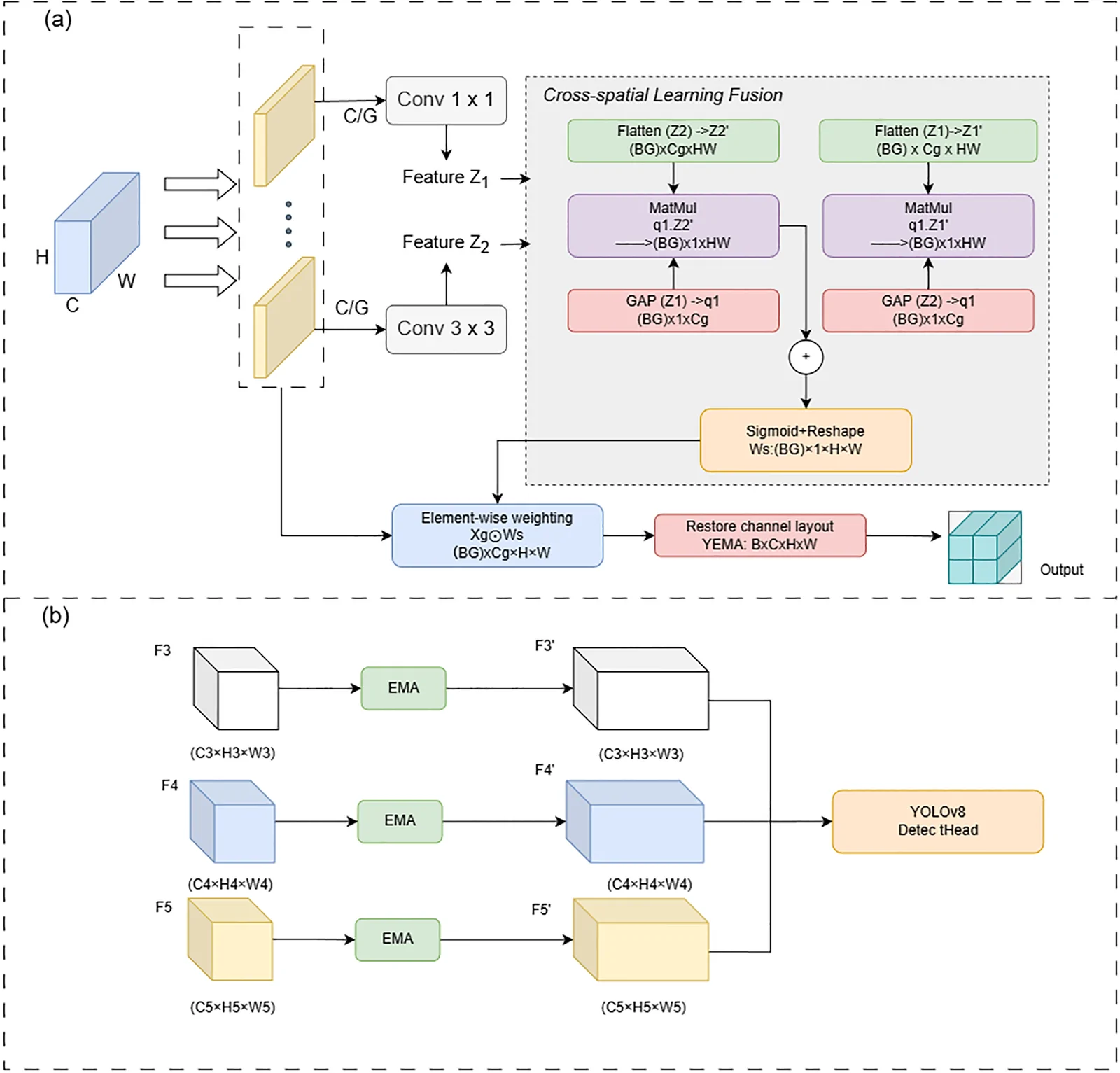
Structure of the proposed Multi-Scale EMA feature recalibration module. (a) Internal structure of the EMA block, which includes group partition, direction-aware attention, local convolution, and normalization-based recalibration. (b) Deployment of EMA blocks on the fused multi-scale features *F*_3_, *F*_4_, and *F*_5_ before the YOLOv8 detection head.

In the proposed RCTE, EMA modules are applied to the fused feature maps at three detection scales. Given the fused multi-scale features *F*_3_, *F*_4_, and *F*_5_, the recalibrated features can be expressed as:


$$\begin{array}{c} {\hat{F}}_{i}=ℰ(F_{i}),\;i\in \{3,4,5\}, \end{array}$$ (7)

where ℰ(·) denotes the EMA operation, $F_{i}$ denotes the fused feature at the corresponding scale, and ${\hat{F}}_{i}$ represents the recalibrated feature after attention enhancement.

As shown in Fig 4(a), the EMA block first divides the input feature into several groups along the channel dimension. This grouped feature representation reduces computational complexity while preserving local channel information. For each grouped feature, adaptive average pooling is performed along the horizontal and vertical directions to capture direction-aware contextual information. The horizontal and vertical descriptors are concatenated and processed by a 1×1 convolution to generate spatial attention weights. In this way, the module can capture long-range dependencies along different spatial directions and enhance the responses of informative regions.

Meanwhile, a local convolution branch is used to preserve local spatial representation. Specifically, a 3×3 convolution is applied to the grouped feature to extract local contextual information. In addition, group normalization is used to stabilize the feature distribution during recalibration. The final output of the EMA module is obtained by combining the spatially enhanced feature and the normalized recalibrated feature:


$$\begin{array}{c} \hat{F}=F_{s1}+F_{s2}, \end{array}$$ (8)

where $F_{s1}$ denotes the spatially enhanced feature generated by direction-aware attention and local convolution, and $F_{s2}$ denotes the recalibrated feature generated from normalized group features and global contextual weights.

Different dental targets rely on different detection scales. Small lesions such as caries and periapical lesions depend more on high-resolution features, while larger anatomical structures such as mandibular canals and maxillary sinuses require stronger semantic features. Therefore, applying EMA to only one feature scale may be insufficient for dental panoramic radiograph detection. As shown in Fig 4(b), EMA modules are applied to *F*_3_, *F*_4_, and *F*_5_ simultaneously, forming a multi-scale feature recalibration strategy. This design enables the model to enhance low-contrast lesions, weak boundaries, and diagnostically important anatomical structures at different scales.

### Implementation details

RCTE-Net was implemented based on the YOLOv8n framework with an input image size of 640×640 pixels. In the model configuration, the feature maps from layers 4, 6, and 9 were selected as *P*_3_, *P*_4_, and *P*_5_, respectively, and were fed into the CSCT module for cross-scale contextual interaction. The enhanced multi-scale features were then separated by feature indexing and passed to the RPF structure for bidirectional feature fusion. After feature fusion, MS-EMA modules were applied to the fused detection features at three scales, and the recalibrated features were sent to the original YOLOv8 detection head for prediction. The original detection head and training objective were retained, while the main improvements of RCTE focused on cross-scale feature interaction, re-parameterized feature fusion, and multi-scale feature recalibration.

### Dataset

The experiments were conducted on the Tooth Filling Detection dataset, a publicly available third-party dental panoramic radiograph dataset released on Roboflow. The dataset is available at https://universe.roboflow.com/pidray-8gl3p/tooth-filling-detection/dataset/2. The dataset consists of de-identified dental panoramic radiographs with multiple annotated dental structures and lesions. The authors did not participate in data collection and did not have access to any personally identifiable patient information.

The dataset contains 11 categories, including Filling, Crown, Root Canal Treatment, Periapical lesion, Missing teeth, Root Piece, Caries, Impacted tooth, Implant, Mandibular Canal, and Maxillary sinus. All annotations were converted into YOLO format for object detection.

In this study, the dataset was divided into training, validation, and test sets with a ratio of 8:1:1. Specifically, 6498 images were used for model training, 813 images were used for validation, and 813 images were used for final testing. The training set was used for model optimization, the validation set was used for monitoring model performance during training, and the test set was used for final performance evaluation. The same dataset split was used for all compared models to ensure a fair comparison. The dataset split is shown in Table 1.

**Table 1:** Dataset split of the Tooth Filling Detection dataset used in this study.

Subset	Number of images	Purpose
Training set	6498	Model training
Validation set	813	Performance monitoring during training
Test set	813	Final model evaluation

### Experimental environment

All experiments were conducted on a local workstation with Windows 11. The hardware platform was equipped with an AMD Ryzen 9 8945HX processor and an NVIDIA GeForce RTX 5060 Laptop GPU with 8 GB of memory. The software environment was based on Python 3.10.0 and PyTorch 2.7.1 with CUDA 12.8 support. The main experimental environment is summarized in Table 2.

**Table 2:** Experimental environment.

Item	Configuration
Operating system	Windows 11
CPU	AMD Ryzen 9 8945HX with Radeon Graphics
GPU	NVIDIA GeForce RTX 5060 Laptop GPU, 8 GB
Programming language	Python 3.10.0
Deep learning framework	PyTorch 2.7.1 + CUDA 12.8
TorchVision	0.22.1 + CUDA 12.8
OpenCV	4.10.0.84
NumPy	2.2.6

### Training settings

To ensure a fair comparison, all models were trained under the same experimental settings. The input image size was set to 640×640, the batch size was set to 16, and the total number of training epochs was set to 100. The SGD optimizer was used for model optimization, with an initial learning rate of 0.01, a final learning rate factor of 0.01, a momentum of 0.937, and a weight decay of 0.0005. The number of data loading workers was set to 0. Automatic mixed precision training was disabled. The IoU threshold for evaluation was set to 0.7, and the maximum number of detections per image was set to 300. The main training settings are listed in Table 3.

**Table 3:** Training settings used in the experiments.

Parameter	Value
Input image size	640×640
Epochs	100
Batch size	16
Optimizer	SGD
Initial learning rate	0.01
Final learning rate factor	0.01
Momentum	0.937
Weight decay	0.0005
Workers	0
AMP	False
IoU threshold	0.7
Maximum detections	300
Annotation format	YOLO format

### Evaluation metrics

To comprehensively evaluate the detection performance and computational efficiency of different models, both accuracy-related and efficiency-related metrics were used in this study. The accuracy-related metrics include Precision (P), Recall (R), F1-score, mAP50, mAP75, and mAP50–95. The efficiency-related metrics include GFLOPs, the number of parameters, preprocessing time, inference time, postprocessing time, FPS, and model file size.

Precision is used to measure the proportion of correctly predicted positive samples among all predicted positive samples, and Recall is used to measure the proportion of correctly detected targets among all ground-truth targets. They are defined as follows:


$$\begin{array}{c} P=\frac{TP}{TP+FP}, \end{array}$$ (9)

$$\begin{array}{c} R=\frac{TP}{TP+FN}, \end{array}$$ (10)

where *TP*, *FP*, and *FN* denote true positives, false positives, and false negatives, respectively. The F1-score is the harmonic mean of Precision and Recall, which reflects the balance between false detections and missed detections:


$$\begin{array}{c} F1=\frac{2PR}{P+R}. \end{array}$$ (11)

The mean average precision (mAP) was used as the main metric for evaluating detection accuracy. In this study, mAP50, mAP75, and mAP50–95 were reported. The mAP50 represents the mean average precision at an IoU threshold of 0.50, while mAP75 represents the mean average precision at a stricter IoU threshold of 0.75. The mAP50–95 calculates the average mAP over multiple IoU thresholds from 0.50 to 0.95 with a step size of 0.05:


$$\begin{array}{c} mAP50\text{--}95=\frac{1}{10}\sum_{t=0.50}^{0.95}AP_{t}, \end{array}$$ (12)

where $AP_{t}$ denotes the average precision at the IoU threshold *t*. Compared with mAP50, mAP75 and mAP50–95 provide a stricter evalua*t*ion of localization accuracy, which is important for dental panoramic radiograph detection because many targets have small sizes, blurred boundaries, and irregular shapes.

In addition to detection accuracy, computational efficiency was also evaluated. GFLOPs were used to measure the theoretical computational complexity of the model, and the number of parameters was used to measure the model scale. The model file size was recorded to evaluate the storage requirement of each model. For inference efficiency, three time-related metrics were reported: preprocessing time, inference time, and postprocessing time per image. The preprocessing time refers to the time required for image resizing and normalization before model inference. The inference time refers to the forward propagation time of the model. The postprocessing time refers to the time required for operations such as confidence filtering and non-maximum suppression.

The inference speed was measured using two FPS metrics. The first one is the overall FPS, which considers preprocessing, model inference, and postprocessing:


$$\begin{array}{c} FPS_{all}=\frac{1}{T_{pre}+T_{infer}+T_{post}}, \end{array}$$ (13)

where $T_{pre}$, $T_{infer}$, and $T_{post}$ denote the preprocessing time, inference time, and postprocessing time for one image, respectively. The second one is the pure inference FPS, which only considers the model inference time:


$$\begin{array}{c} FPS_{infer}=\frac{1}{T_{infer}}. \end{array}$$ (14)

For the dental panoramic radiograph detection task, mAP50–95, Recall, mAP75, and F1-score were regarded as the most important indicators. The mAP50–95 reflects the overall detection and localization performance under multiple IoU thresholds. Recall is particularly important because missed detections may affect subsequent clinical interpretation. The mAP75 reflects the localization ability under a stricter IoU threshold, and the F1-score evaluates the balance between Precision and Recall. Precision, FPS, parameters, GFLOPs, and model file size were used as auxiliary indicators to analyze false detections and computational cost.

### Comparison with baseline detectors

To verify the effectiveness of the proposed RCTE, several representative YOLO-based detectors were selected for comparison, including YOLOv5n, YOLOv6n, YOLOv8n, YOLOv11n, and YOLO26n. These models represent different stages of YOLO-based detector development and provide different accuracy-efficiency trade-offs. All models were trained and evaluated on the same Tooth Filling Detection dataset under the same training settings. The comparison results are shown in Table 4.

**Table 4:** Comparison with different YOLO-based detectors on the Tooth Filling Detection dataset.

Model	Params	FPS	P	R	F1	mAP50	mAP75	mAP50–95
YOLOv5n	2.18M	267.18	0.5650	0.5860	0.5574	0.5570	0.3141	0.3091
YOLOv6n	4.16M	273.22	0.5373	0.5983	0.5434	0.5435	0.3175	0.3071
YOLOv8n	2.69M	**478.78**	0.5501	0.5562	0.5367	0.5450	0.3065	0.3036
YOLOv11n	2.58M	405.16	0.5395	0.5578	0.5320	0.5330	0.3116	0.3006
YOLO26n	**2.50M**	422.98	**0.7072**	0.4707	0.4714	0.5206	0.2589	0.2754
**RCTE-Net**	5.46M	202.34	0.5766	**0.6072**	**0.5696**	**0.5705**	**0.3453**	**0.3276**

As shown in Table 4, RCTE achieves the best Recall, F1-score, mAP50, mAP75, and mAP50–95 among all compared models. Compared with the baseline YOLOv8n, RCTE improves Recall, F1-score, mAP50, mAP75, and mAP50–95 by 5.10, 3.29, 2.55, 3.88, and 2.40 percentage points, respectively. These improvements indicate that the proposed network effectively enhances the detection completeness and localization accuracy of dental targets.

Compared with YOLOv5n, RCTE improves mAP50, mAP75, and mAP50–95 by 1.35, 3.12, and 1.85 percentage points, respectively. Although YOLOv5n already shows competitive performance on this dataset, RCTE still achieves higher detection accuracy, especially under the stricter mAP75 metric. This demonstrates that the proposed cross-scale interaction and re-parameterized feature fusion are beneficial for improving localization accuracy in dental panoramic radiographs.

YOLOv6n obtains a relatively high Recall of 0.5983, but its Precision, F1-score, and mAP metrics are lower than those of RCTE. This indicates that YOLOv6n can detect more targets than some baseline models but still suffers from insufficient localization and classification accuracy. In contrast, RCTE achieves a better balance between Recall and Precision, resulting in the highest F1-score and mAP50–95.

YOLOv11n and YOLO26n have fewer parameters and higher inference speeds than RCTE. However, their detection accuracy is lower. In particular, YOLO26n achieves the highest Precision of 0.7072, but its Recall is only 0.4707, indicating that it tends to make conservative predictions and suffers from missed detections. For dental panoramic radiograph analysis, missed detections of lesions or important anatomical structures may reduce the reliability of computer-aided diagnosis. Therefore, Recall, F1-score, and mAP50–95 are more important than Precision alone. Compared with YOLO26n, RCTE improves Recall, F1-score, mAP50, mAP75, and mAP50–95 by 13.65, 9.82, 4.99, 8.64, and 5.22 percentage points, respectively.

Although RCTE introduces additional parameters and reduces inference speed compared with lightweight baseline models, it still achieves an inference speed of 202.34 FPS, which indicates that the proposed method maintains real-time detection capability. Therefore, RCTE provides a favorable trade-off between detection accuracy and computational efficiency for multi-class object detection in dental panoramic radiographs.

### Qualitative comparison with baseline detectors

To further intuitively evaluate the detection performance of different models, qualitative visualization results are presented in Fig 5. Several representative dental panoramic radiographs were selected from the test set. The compared methods include YOLOv5n, YOLOv6n, YOLOv8n, and the proposed RCTE.

**Fig 5:**
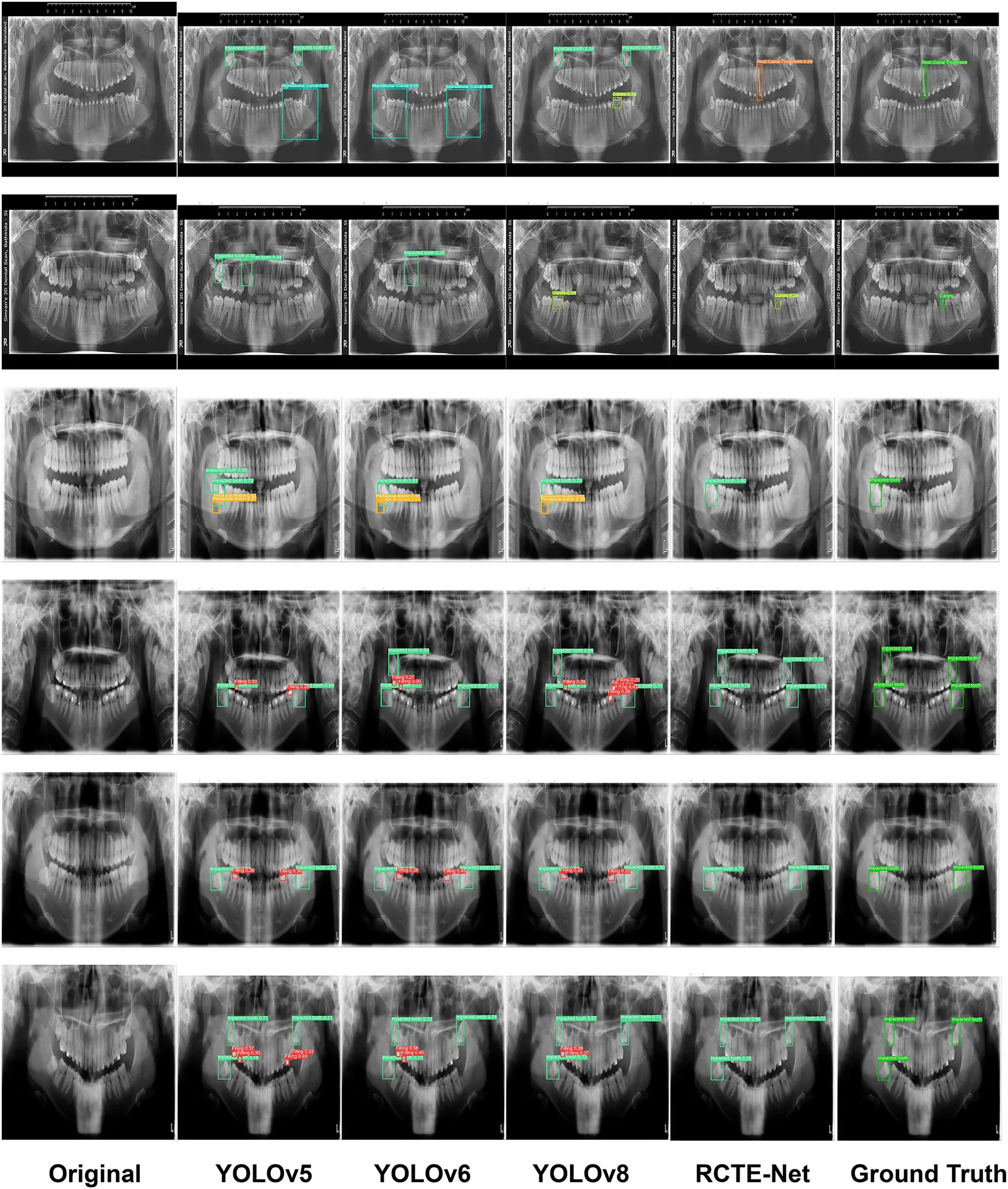
Qualitative comparison with baseline detectors on the Tooth Filling Detection dataset. From left to right: original image, YOLOv5n, YOLOv6n, YOLOv8n, RCTE, and ground truth.

As shown in Fig 5, the baseline detectors still suffer from missed detections, false-positive predictions, and inaccurate localization in complex dental panoramic radiographs. YOLOv5n and YOLOv6n may produce redundant predictions on adjacent anatomical structures, while YOLOv8n still shows unstable responses for small lesions and weak-boundary targets. In contrast, RCTE provides detection results that are more consistent with the ground-truth annotations. The proposed model can better suppress false-positive responses and improve the localization of dental targets with ambiguous boundaries. These qualitative results are consistent with the quantitative comparison in Table 4, further demonstrating the effectiveness of the proposed cross-scale contextual interaction, re-parameterized feature fusion, and multi-scale feature recalibration.

Fig 6 provides an additional qualitative comparison with YOLOv11n and YOLO26n. Although these models can detect several obvious targets, they still show missed detections or redundant predictions in challenging cases. RCTE achieves more consistent detection results, which further supports the quantitative improvements in Recall, F1-score, mAP75, and mAP50–95.

**Fig 6:**
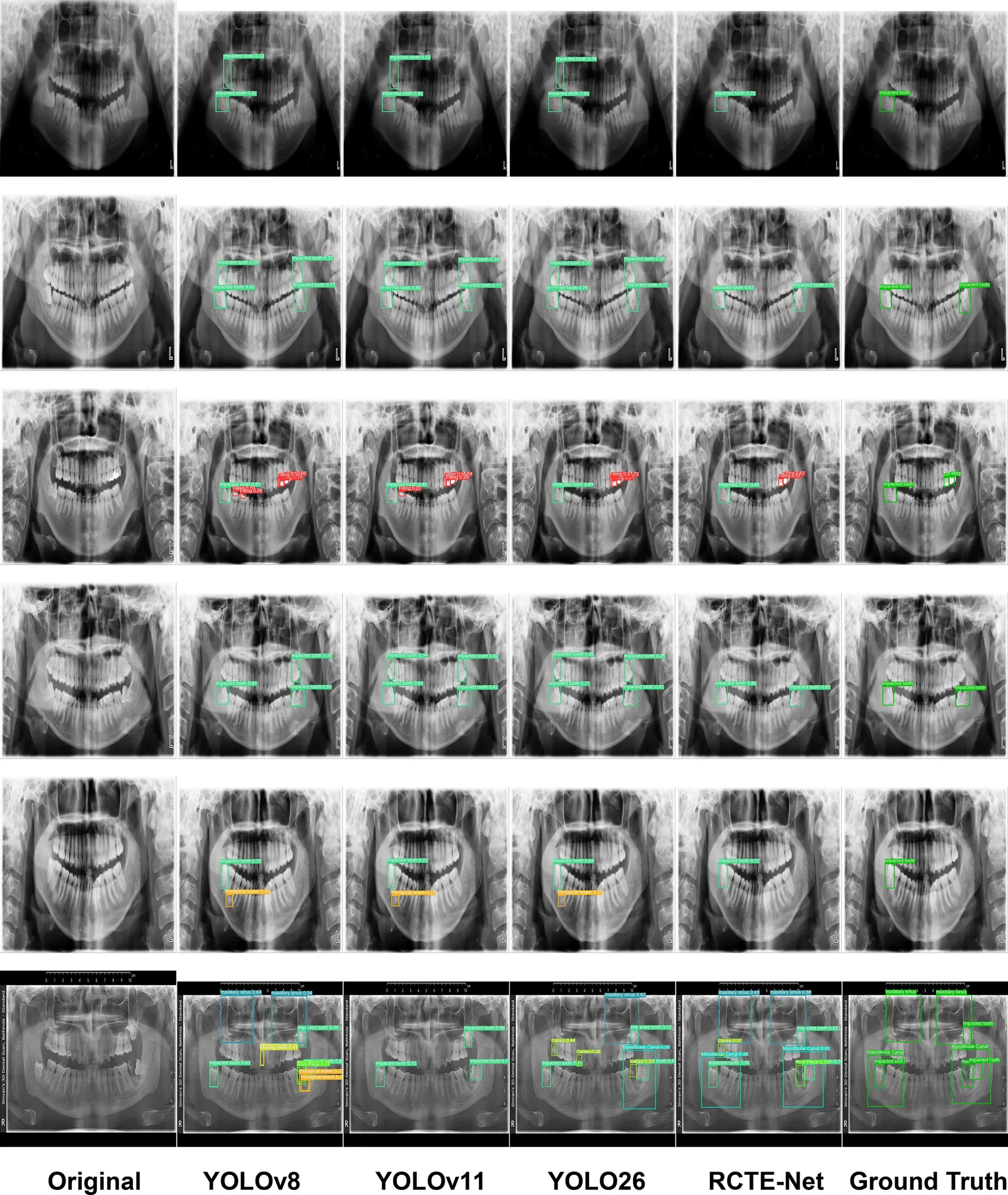
Additional qualitative comparison with recent YOLO-based detectors. From left to right: original image, YOLOv8n, YOLOv11n, YOLO26n, RCTE, and ground truth.

## Model performance analysis

### Training convergence analysis

To analyze the training process and category-level detection behavior of RCTE, the training curves, Precision-Recall curve, and normalized confusion matrix are presented.

The training and validation curves of RCTE are shown in Fig 7. The training box loss, classification loss, and distribution focal loss decrease rapidly during the early training stage and gradually converge as the number of epochs increases. A similar decreasing trend can also be observed in the validation losses, indicating effective feature learning without obvious divergence. The mAP50 and mAP50–95 curves increase continuously in the early and middle training stages and stabilize later. The Recall curve rises quickly at the beginning and remains relatively stable, demonstrating that RCTE has stable convergence behavior on the Tooth Filling Detection dataset.

**Fig 7:**
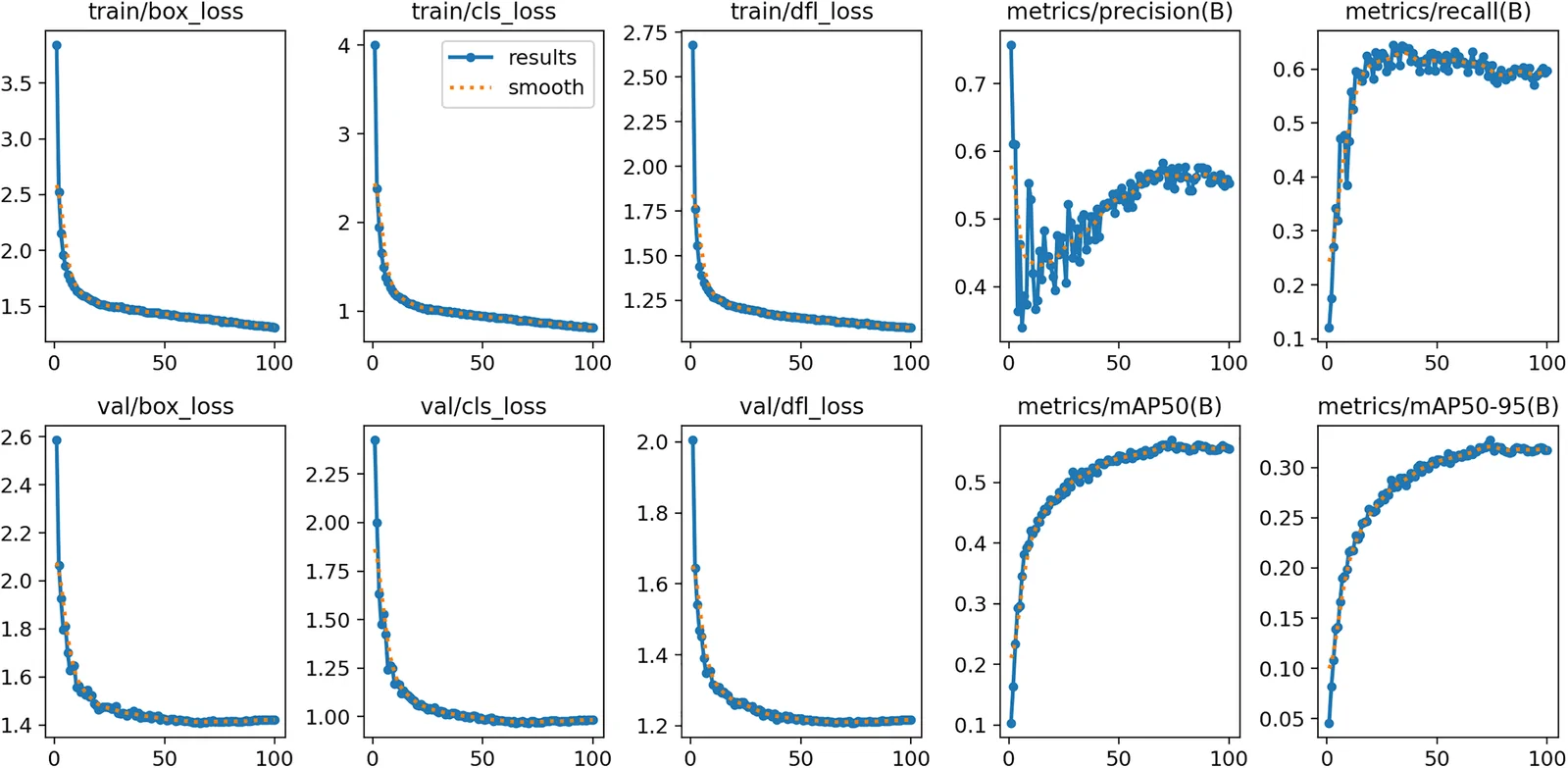
Training and validation curves of RCTE. The losses gradually decrease, while Precision, Recall, mAP50, and mAP50–95 become stable during training.

### Precision-recall curve

The Precision-Recall curve of RCTE is shown in Fig 8. The overall mAP50 reaches 0.570, and clear differences can be observed among different categories. Implant and impacted tooth achieve high AP values of 0.920 and 0.951, respectively, indicating these categories have relatively distinctive visual patterns. Crown also obtains a relatively high AP of 0.797, whereas periapical lesion and caries show lower AP values due to small sizes, weak contrast, and ambiguous boundaries.

**Fig 8:**
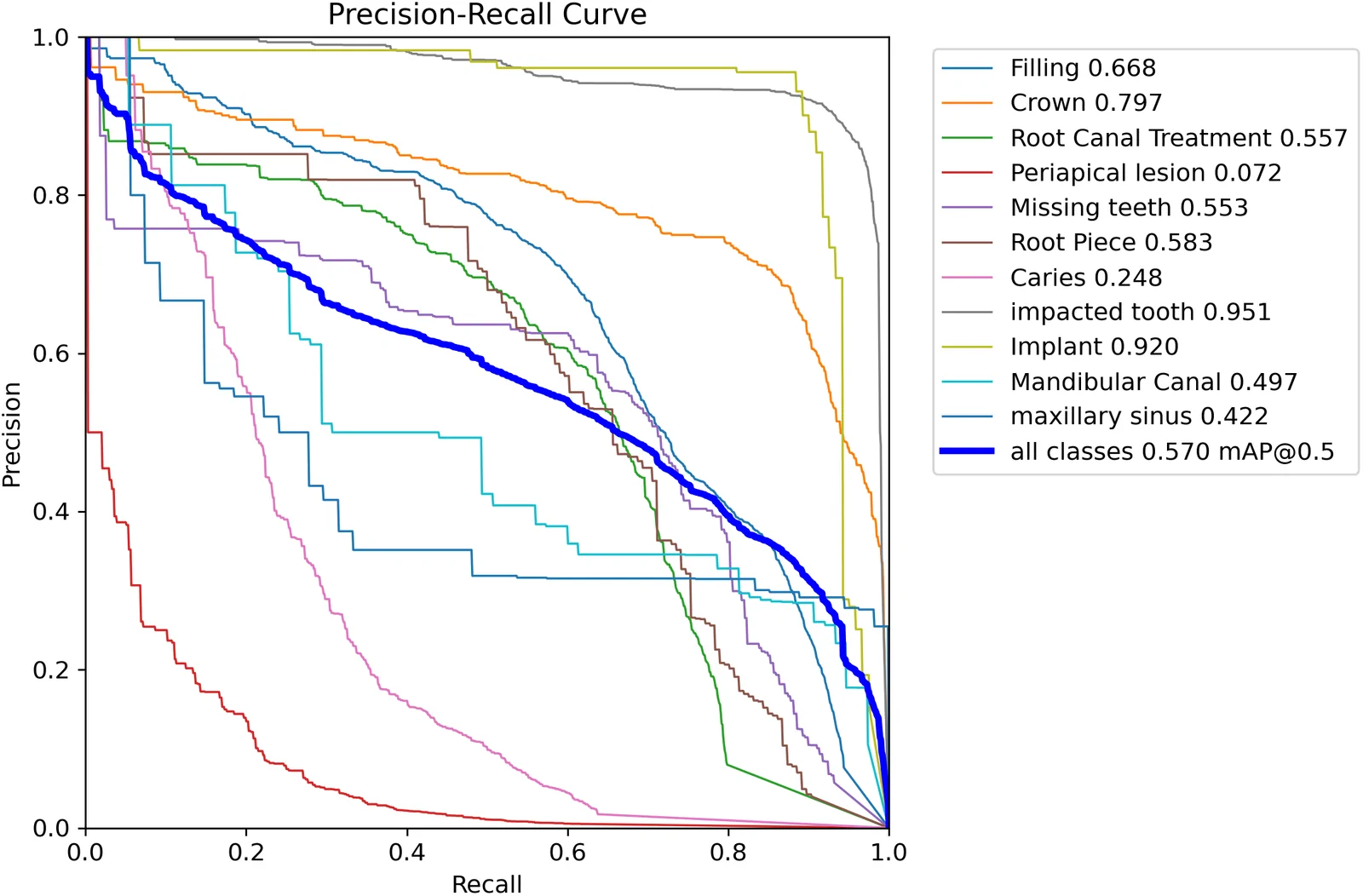
Precision-Recall curve of RCTE on the Tooth Filling Detection dataset. The proposed model achieves an overall mAP50 of 0.570.

### Per-class detection performance

Table 5 shows the per-class detection performance of RCTE on the Tooth Filling Detection dataset. Strong detection performance is observed for impacted tooth, Implant, and Crown. Periapical lesion and caries remain challenging, consistent with the PR curve.

**Table 5:** Per-class detection performance of RCTE on the Tooth Filling Detection dataset.

Class	P	R	F1	mAP50	mAP75	mAP50–95
Filling	0.6654	0.6287	0.6465	0.6684	0.3676	0.3697
Crown	0.6949	0.8629	0.7699	0.7964	0.7183	0.5757
Root Canal Treatment	0.6212	0.5786	0.5991	0.5632	0.1709	0.2379
Periapical lesion	0.2852	0.0685	0.1104	0.0726	0.0063	0.0208
Missing teeth	0.5550	0.6619	0.6037	0.5537	0.1702	0.2458
Root Piece	0.5053	0.6506	0.5688	0.5776	0.1983	0.2621
Caries	0.5618	0.1998	0.2948	0.2497	0.0453	0.0904
Impacted tooth	0.8673	0.9599	0.9113	0.9513	0.7679	0.6407
Implant	0.8845	0.9091	0.8966	0.9203	0.6739	0.5576
Mandibular Canal	0.3998	0.5333	0.4570	0.4968	0.3179	0.2845
Maxillary sinus	0.3024	0.6263	0.4079	0.4257	0.3622	0.3188
Average	0.5766	0.6072	0.5696	0.5705	0.3453	0.3276

### Normalized confusion matrix

The normalized confusion matrix on the test set is shown in Fig 9. Many difficult samples, especially periapical lesion and caries, are confused with the background. This suggests missed detections are mainly caused by weak lesion contrast and unclear boundaries rather than severe confusion among foreground categories.

**Fig 9:**
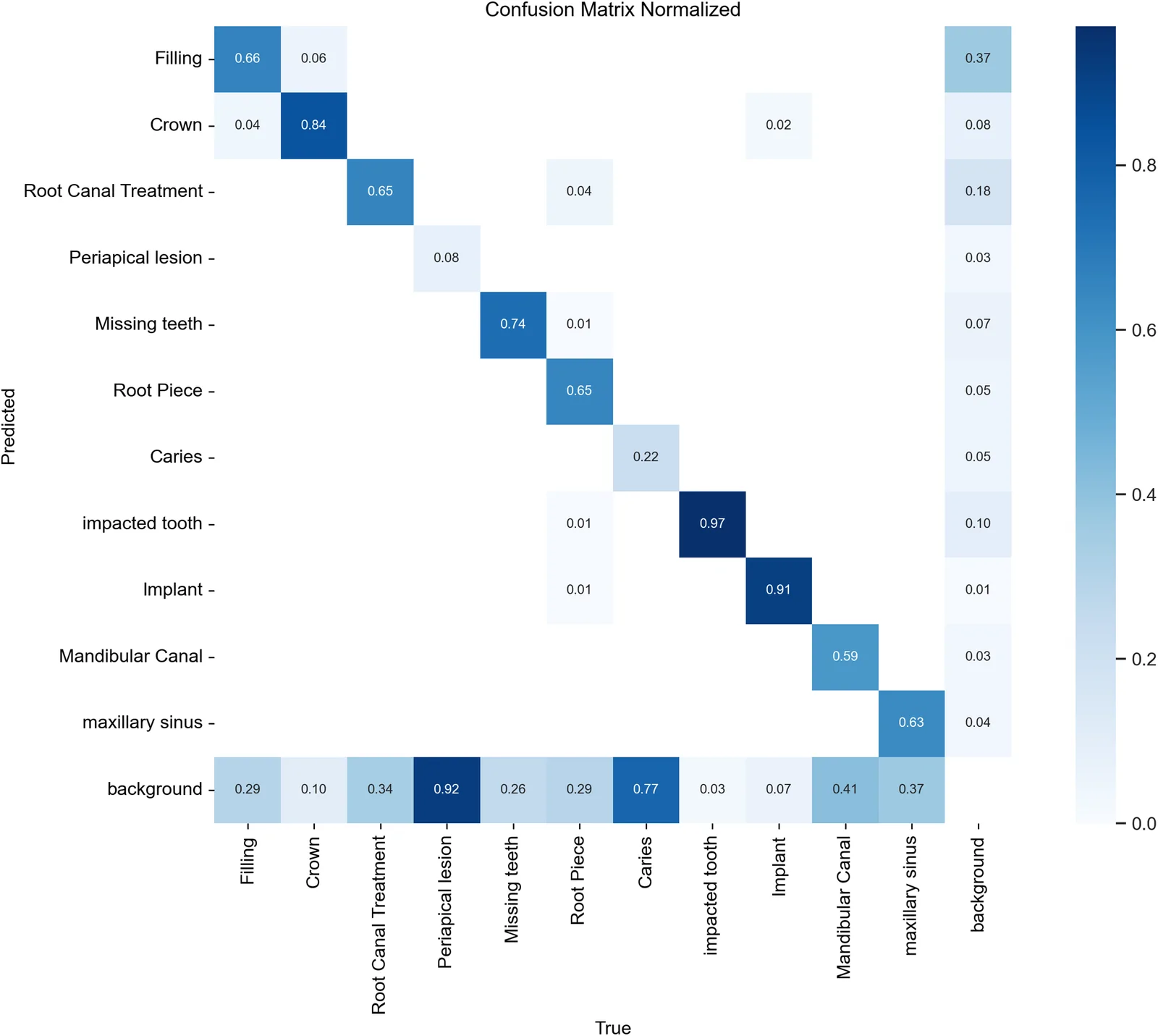
Normalized confusion matrix of RCTE on the test set. The matrix shows classification performance and error distribution among different dental categories.

### Ablation study

To verify the effectiveness of the proposed modules, ablation experiments were conducted on the Tooth Filling Detection dataset. YOLOv8n was used as the baseline. The CSCT, RPF, and MS-EMA modules were added separately and jointly. The results are shown in Table 6.

**Table 6:** Ablation study of the proposed modules on the tooth filling detection dataset.

CSCT	RPF	MS-EMA	Params	FPS	P	R	F1	mAP50	mAP75	mAP50–95
–	–	–	2.69M	**478.78**	0.5501	0.5562	0.5367	0.5450	0.3065	0.3036
✓	–	–	5.64M	350.96	0.5467	0.6153	0.5602	0.5460	0.3200	0.3088
–	✓	–	2.83M	315.09	**0.5785**	**0.6181**	**0.5728**	0.5611	0.3348	0.3210
–	–	✓	3.01M	353.11	0.5620	0.6107	0.5671	0.5517	0.3127	0.3093
✓	✓	✓	5.46M	202.34	0.5766	0.6072	0.5696	**0.5705**	**0.3453**	**0.3276**

The first row denotes the original YOLOv8n baseline. The last row denotes the complete RCTE. The best result for each metric is shown in bold. FPS denotes pure inference FPS.

### Per-class ablation mAP75

Table 7 shows the per-class mAP75 for the ablation variants. RCTE achieves the highest average mAP75, demonstrating complementary effects of CSCT, RPF, and MS-EMA.

**Table 7:** Per-class mAP75 comparison of different ablation variants.

Class	YOLOv8n	+CSCT	+RPF	+MS-EMA	RCTE
Filling	0.3605	0.3696	**0.3771**	0.3522	0.3676
Crown	0.6922	0.6848	**0.7297**	0.6743	0.7183
Root Canal Treatment	0.1511	0.1654	0.1574	0.1686	**0.1709**
Periapical lesion	0.0053	**0.0084**	0.0039	0.0030	0.0063
Missing teeth	0.1488	**0.1990**	0.1497	0.1560	0.1702
Root Piece	0.1864	**0.1988**	0.1824	0.1448	0.1983
Caries	0.0336	0.0350	0.0418	0.0355	**0.0453**
Impacted tooth	**0.7685**	0.7601	0.7599	0.7586	0.7679
Implant	0.5320	0.6339	0.6387	0.6502	**0.6739**
Mandibular Canal	0.2284	0.1453	0.2789	0.2391	**0.3179**
Maxillary sinus	0.2644	0.3200	**0.3628**	0.2579	0.3622
Average	0.3065	0.3200	0.3348	0.3127	**0.3453**

### Qualitative visualization of ablation results

Qualitative visualization of the ablation study is shown in Fig 10. The original YOLOv8n baseline tends to generate false-positive predictions or incomplete detections. After introducing CSCT, detection completeness improves. The MS-EMA module further recalibrates multi-scale features, enhancing responses to diagnostically relevant regions. The RPF structure strengthens feature fusion and localization. The complete RCTE produces cleaner and more accurate detection results, showing that the three modules are complementary.

**Fig 10:**
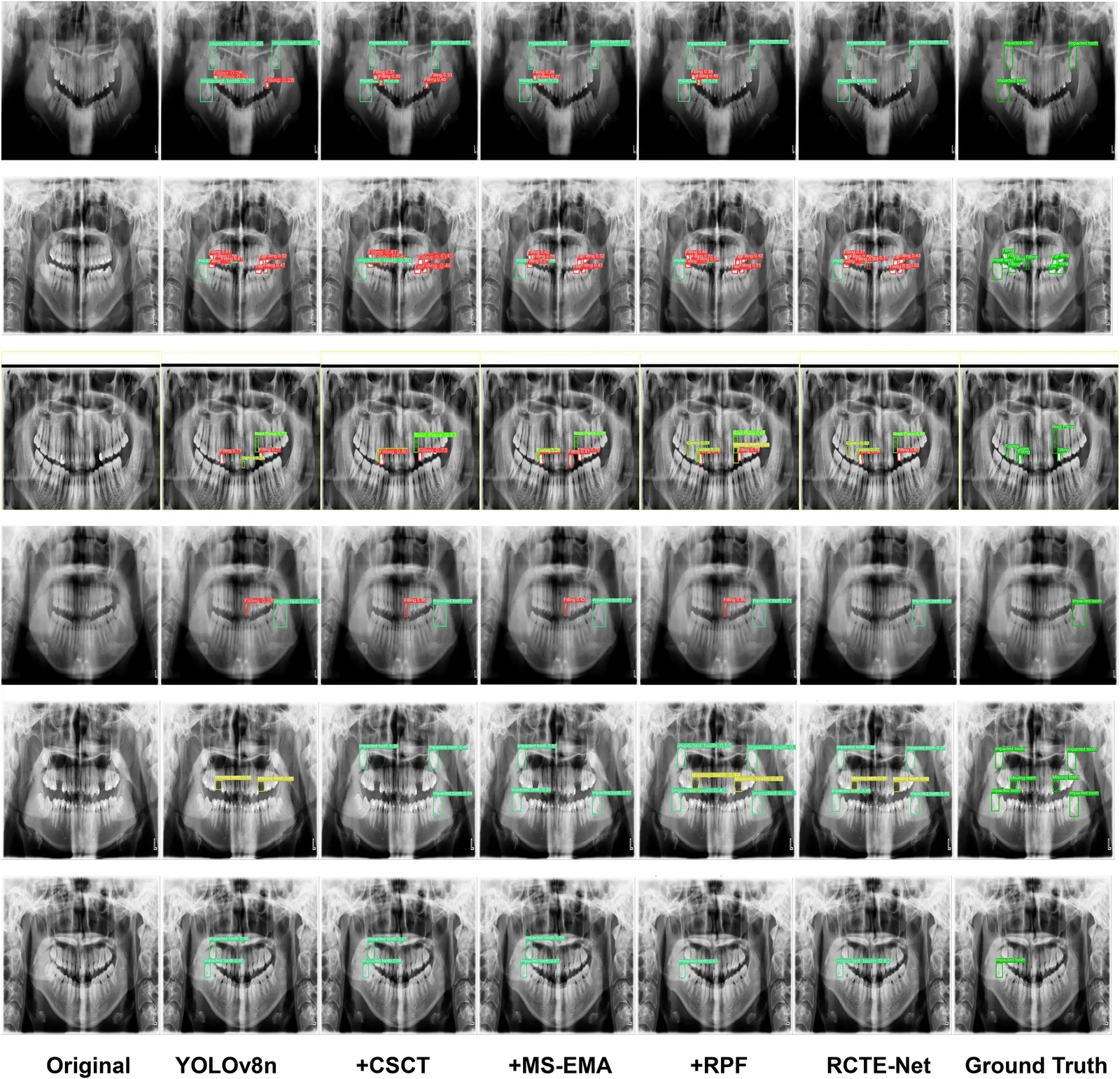
Qualitative visualization results of the ablation study. From left to right: original image, YOLOv8n, YOLOv8n+CSCT, YOLOv8n+MS-EMA, YOLOv8n+RPF, RCTE, and ground truth.

## Discussion

The experimental results demonstrate that RCTE achieves superior overall detection performance compared with representative YOLO-based detectors on the dental panoramic radiograph dataset. Compared with the original YOLOv8n baseline, RCTE improves mAP50, mAP75, mAP50–95, and Recall by 2.55, 3.88, 2.40, and 5.10 percentage points, respectively. These improvements indicate that the proposed framework enhances both detection completeness and localization accuracy for multi-class dental object detection. Recent studies have also demonstrated the potential of artificial intelligence-based methods for automated assessment of multiple dental findings from panoramic radiographs, supporting the clinical relevance of automated dental image analysis [21].

The improved performance of RCTE can be attributed to the complementary effects of cross-scale contextual modeling, multi-scale feature fusion, and feature recalibration. The ablation experiments demonstrate that each proposed component contributes to performance improvement, while their combination achieves the best overall detection capability. In particular, the enhanced localization performance indicates that effective feature representation and multi-scale information aggregation are beneficial for identifying dental targets with different scales and complex anatomical backgrounds. Similar challenges have been reported in medical image analysis, where variations in object size, unclear boundaries, and complex surrounding structures remain important factors affecting detection accuracy [22].

The category-level analysis shows that RCTE performs well on relatively distinctive structures, including Impacted tooth, Implant, and Crown. However, Caries and Periapical lesion remain challenging categories due to their small size, low contrast, irregular appearance, and similarity to surrounding tissues. Previous studies on dental lesion detection have also reported that these categories are difficult to identify accurately on panoramic radiographs because of limited visual information and complex anatomical contexts [23,24]. In addition, differences in category distribution and annotation difficulty may further influence the detection performance of challenging classes. Future improvements may explore adaptive sampling strategies and loss optimization methods, such as focal loss, to further enhance the recognition capability of difficult categories [25].

Compared with other YOLO-based detectors, RCTE achieves better overall detection performance, especially in Recall and mAP-based metrics. For dental panoramic radiograph analysis, reducing missed detections is particularly important because undetected lesions or anatomical abnormalities may affect subsequent clinical assessment. Although RCTE introduces additional computational overhead, it maintains real-time inference capability with an inference speed of 202.34 FPS. This demonstrates a reasonable balance between detection accuracy and computational efficiency, which is consistent with recent studies focusing on lightweight and efficient dental detection frameworks [26,27].

Despite the promising results, several limitations remain. First, although the dataset contains multiple dental categories, further external validation using larger multi-center datasets is required to evaluate the generalization capability of RCTE. Second, the current framework mainly focuses on two-dimensional panoramic radiographs, while some anatomical structures, such as the mandibular canal, may benefit from complementary three-dimensional information in complex clinical scenarios [28]. Third, more comprehensive clinical evaluation and deployment studies are required before practical application. Future work will focus on improving the detection of small lesions, validating the model on more diverse datasets, and optimizing the framework for practical clinical deployment.

## Conclusions

This study proposed RCTE, a re-parameterized cross-scale attention-enhanced framework for multi-class object detection in dental panoramic radiographs. By integrating cross-scale contextual interaction, re-parameterized feature fusion, and multi-scale feature recalibration, RCTE improves the representation capability of complex dental targets.

Experimental results demonstrate that RCTE achieves superior detection performance compared with YOLOv8n and other YOLO-based baselines, particularly in terms of recall and localization accuracy. The proposed framework provides an effective approach for computer-aided dental image analysis.

However, consistent with the broader challenges of artificial intelligence in medical imaging, further improvements require larger datasets, external validation, transparent reporting, and clinical deployment assessment [29–32]. Future studies will focus on improving the detection of small and low-contrast lesions, evaluating the model on larger multi-center datasets, and optimizing the framework for practical clinical applications.

## References

[1] Hamamci IE, Er S, Durugol OF, Cakmak GR, de la Rosa E, Simsar E. Dentex: Dental enumeration and tooth pathosis detection benchmark for panoramic x-ray. arXiv. 2025. doi: 10.48550/arXiv.2305.19112

[2] Kuwana R, Ariji Y, Fukuda M, Kise Y, Nozawa M, Kuwada C, et al. Performance of deep learning object detection technology in the detection and diagnosis of maxillary sinus lesions on panoramic radiographs. Dentomaxillofac Radiol. 2021;50(1):20200171. doi: 10.1259/dmfr.20200171 32618480 PMC7780831

[3] Ariji Y, Yanashita Y, Kutsuna S, Muramatsu C, Fukuda M, Kise Y, et al. Automatic detection and classification of radiolucent lesions in the mandible on panoramic radiographs using a deep learning object detection technique. Oral Surg Oral Med Oral Pathol Oral Radiol. 2019;128(4):424–30. doi: 10.1016/j.oooo.2019.05.014 31320299

[4] Beser B, Reis T, Berber MN, Topaloglu E, Gungor E, Kılıc MC, et al. YOLO-V5 based deep learning approach for tooth detection and segmentation on pediatric panoramic radiographs in mixed dentition. BMC Med Imaging. 2024;24(1):172. doi: 10.1186/s12880-024-01338-w 38992601 PMC11238494

[5] Tassoker M, Öziç MÜ, Yuce F. Performance evaluation of a deep learning model for automatic detection and localization of idiopathic osteosclerosis on dental panoramic radiographs. Sci Rep. 2024;14(1):4437. doi: 10.1038/s41598-024-55109-2 38396289 PMC10891049

[6] Yaseen M. What is YOLOv8: An in-depth exploration of the internal features of the next-generation object detector. 2024. doi: 10.48550/arXiv.2408.15857

[7] Wang CY, Yeh IH, Liao HYM. YOLOv9: Learning What You Want to Learn Using Programmable Gradient Information. Computer Vision – ECCV 2024. 2025. p. 1–21. doi: 10.1007/978-3-031-72751-1_1

[8] Ouyang D, He S, Zhang G, Luo M, Guo H, Zhan J, et al. Efficient Multi-Scale Attention Module with Cross-Spatial Learning. In: ICASSP 2023 - 2023 IEEE International Conference on Acoustics, Speech and Signal Processing (ICASSP), 2023. 1–5. 10.1109/icassp49357.2023.10096516

[9] Lin T-Y, Dollar P, Girshick R, He K, Hariharan B, Belongie S. Feature Pyramid Networks for Object Detection. In: 2017 IEEE Conference on Computer Vision and Pattern Recognition (CVPR), 2017. 936–44. 10.1109/cvpr.2017.106

[10] Woo S, Park J, Lee JY, Kweon IS. CBAM: Convolutional Block Attention Module. Computer Vision – ECCV 2018. 2018. p. 3–19. doi: 10.1007/978-3-030-01234-2_1

[11] Tuzoff DV, Tuzova LN, Bornstein MM, Krasnov AS, Kharchenko MA, Nikolenko SI, et al. Tooth detection and numbering in panoramic radiographs using convolutional neural networks. Dentomaxillofac Radiol. 2019;48(4):20180051. doi: 10.1259/dmfr.20180051 30835551 PMC6592580

[12] Lee J-H, Kim D-H, Jeong S-N, Choi S-H. Detection and diagnosis of dental caries using a deep learning-based convolutional neural network algorithm. J Dent. 2018;77:106–11. doi: 10.1016/j.jdent.2018.07.015 30056118

[13] Lee S, Oh S-I, Jo J, Kang S, Shin Y, Park J-W. Deep learning for early dental caries detection in bitewing radiographs. Sci Rep. 2021;11(1):16807. doi: 10.1038/s41598-021-96368-7 34413414 PMC8376948

[14] Mohammad-Rahimi H, Motamedian SR, Rohban MH, Krois J, Uribe SE, Mahmoudinia E, et al. Deep learning for caries detection: A systematic review. J Dent. 2022;122:104115. doi: 10.1016/j.jdent.2022.104115 35367318

[15] Shafiq M, Gu Z. Deep Residual Learning for Image Recognition: A Survey. Applied Sciences. 2022;12(18):8972. doi: 10.3390/app12188972

[16] Liu S, Qi L, Qin H, Shi J, Jia J. Path Aggregation Network for Instance Segmentation. In: 2018 IEEE/CVF Conference on Computer Vision and Pattern Recognition, 2018. 8759–68. 10.1109/cvpr.2018.00913

[17] Tan M, Pang R, Le QV. EfficientDet: Scalable and Efficient Object Detection. In: 2020 IEEE/CVF Conference on Computer Vision and Pattern Recognition (CVPR), 2020. 10778–87. 10.1109/cvpr42600.2020.01079

[18] Vaswani A, Shazeer N, Parmar N, Uszkoreit J, Jones L, Gomez AN. Attention Is All You Need. In: 2017. 10.48550/arXiv.1706.03762

[19] Hu J, Shen L, Sun G. Squeeze-and-Excitation Networks. In: 2018 IEEE/CVF Conference on Computer Vision and Pattern Recognition, 2018. 7132–41. 10.1109/cvpr.2018.00745

[20] Hou Q, Zhou D, Feng J. Coordinate Attention for Efficient Mobile Network Design. In: 2021 IEEE/CVF Conference on Computer Vision and Pattern Recognition (CVPR), 2021. 13708–17. 10.1109/cvpr46437.2021.01350

[21] Wang YCC, Chen TL, Vinayahalingam S, Wu TH, Chang CW, Chang HH, et al. Artificial Intelligence to Assess Dental Findings from Panoramic Radiographs – A Multinational Study; 2025. Available from: doi: 10.48550/arXiv.2502.10277

[22] Albuquerque C, Henriques R, Castelli M. Deep learning-based object detection algorithms in medical imaging: Systematic review. Heliyon. 2024;11(1):e41137. doi: 10.1016/j.heliyon.2024.e41137 39758372 PMC11699422

[23] Szabó V, Orhan K, Dobó-Nagy C, Veres DS, Manulis D, Ezhov M, et al. Deep Learning-Based Periapical Lesion Detection on Panoramic Radiographs. Diagnostics. 2025;15(4):510. doi: 10.3390/diagnostics15040510 PMC11854226 40002661

[24] Ba-Hattab R, Barhom N, Osman S, Naceur I, Odeh A, Asad A, et al. Detection of Periapical Lesions on Panoramic Radiographs Using Deep Learning. Applied Sciences. 2023;13(3):1516. doi: 10.3390/app13031516

[25] Lin T-Y, Goyal P, Girshick R, He K, Dollar P. Focal Loss for Dense Object Detection. In: 2017 IEEE International Conference on Computer Vision (ICCV), 2017. 2999–3007. 10.1109/iccv.2017.324

[26] Hua Y, Chen R, Qin H. YOLO-DentSeg: A Lightweight Real-Time Model for Accurate Detection and Segmentation of Oral Diseases in Panoramic Radiographs. Electronics. 2025;14(4):805. doi: 10.3390/electronics14040805

[27] Peker RB, Kurtoglu CO. Evaluation of the Performance of a YOLOv10-Based Deep Learning Model for Tooth Detection and Numbering on Panoramic Radiographs of Patients in the Mixed Dentition Period. Diagnostics (Basel). 2025;15(4):405. doi: 10.3390/diagnostics15040405 40002557 PMC11854638

[28] Aung MTZ, Lim S-H, Han J, Yang S, Kang J-H, Kim J-E, et al. Deep learning-based automatic segmentation of the mandibular canal on panoramic radiographs: A multi-device study. Imaging Sci Dent. 2024;54(1):81–91. doi: 10.5624/isd.20230245 38571772 PMC10985527

[29] Mongan J, Moy L, Kahn CE Jr. Checklist for Artificial Intelligence in Medical Imaging (CLAIM): A Guide for Authors and Reviewers. Radiol Artif Intell. 2020;2(2):e200029. doi: 10.1148/ryai.2020200029 33937821 PMC8017414

[30] Kelly CJ, Karthikesalingam A, Suleyman M, Corrado G, King D. Key challenges for delivering clinical impact with artificial intelligence. BMC Med. 2019;17(1):195. doi: 10.1186/s12916-019-1426-2 31665002 PMC6821018

[31] Collins GS, Moons KGM, Dhiman P, Riley RD, Beam AL, Van Calster B, et al. TRIPOD+AI statement: updated guidance for reporting clinical prediction models that use regression or machine learning methods. BMJ. 2024;385:e078378. doi: 10.1136/bmj-2023-078378 38626948 PMC11019967

[32] Litjens G, Kooi T, Bejnordi BE, Setio AAA, Ciompi F, Ghafoorian M, et al. A survey on deep learning in medical image analysis. Med Image Anal. 2017;42:60–88. doi: 10.1016/j.media.2017.07.005 28778026

